# Approaching Ultimate Synthesis Reaction Rate of Ni-Rich Layered Cathodes for Lithium-Ion Batteries

**DOI:** 10.1007/s40820-024-01436-y

**Published:** 2024-06-06

**Authors:** Zhedong Liu, Jingchao Zhang, Jiawei Luo, Zhaoxin Guo, Haoran Jiang, Zekun Li, Yuhang Liu, Zijing Song, Rui Liu, Wei-Di Liu, Wenbin Hu, Yanan Chen

**Affiliations:** 1 School of Materials Science and Engineering, Tianjin University, Tianjin, 300072 People’s Republic of China; 2 School of Materials Science and Engineering, Shandong University of Science and Technology, Qingdao, 266590 People’s Republic of China; 3 Australian Institute of Bioengineering and Nanotechnology, The University of Queensland, St Lucia, QLD 4072 Australia

**Keywords:** Nickel-rich layered oxides, High-temperature shock, Solid reaction kinetics, Phase transition, Reaction rate

## Abstract

Nickel-rich layered oxide LiNi_x_Co_y_Mn_z_O_2_ (NCM, x + y + z = 1) is the most promising cathode material for high-energy lithium-ion batteries. However, conventional synthesis methods are limited by the slow heating rate, sluggish reaction dynamics, high energy consumption, and long reaction time. To overcome these challenges, we first employed a high-temperature shock (HTS) strategy for fast synthesis of the NCM, and the approaching ultimate reaction rate of solid phase transition is deeply investigated for the first time. In the HTS process, ultrafast average reaction rate of phase transition from Ni_0.6_Co_0.2_Mn_0.2_(OH)_2_ to Li- containing oxides is 66.7 (% s^−1^), that is, taking only 1.5 s. An ultrahigh heating rate leads to fast reaction kinetics, which induces the rapid phase transition of NCM cathodes. The HTS-synthesized nickel-rich layered oxides perform good cycling performances (94% for NCM523, 94% for NCM622, and 80% for NCM811 after 200 cycles at 4.3 V). These findings might also assist to pave the way for preparing effectively Ni-rich layered oxides for lithium-ion batteries.

**Supplementary Information:**

The online version contains supplementary material available at 10.1007/s40820-024-01436-y.

## Highlights

- A series of layered oxide cathode materials were synthesized by high-temperature shock strategy for the first time.
- The approaching ultimate solid reaction rate of the layered nickel-rich layered oxide LiNi_x_Co_y_Mn_z_O_2_ was investigated for the first time. Ultrafast average reaction rate of phase transition from Ni_0.6_Co_0.2_Mn_0.2_(OH)_2_ to Li-containing oxides is 66.7 (% s^-1^), that is, taking only 1.5 s.

**Supplementary Information:**

The online version contains supplementary material available at 10.1007/s40820-024-01436-y.

## Graphical abstract

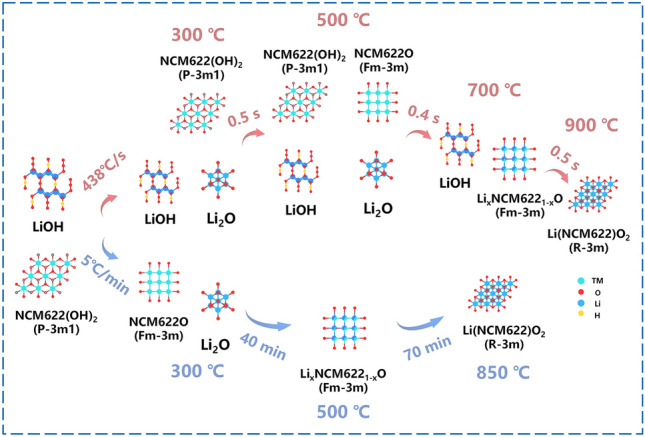

## Introduction

Lithium-ion batteries (LIBs) are desired electricity sources for transportable digital units and are growing, being adopted for electric-powered automobiles and grid storage markets [1–4]. However, low energy density of LIBs has significantly obstructed the widespread application of electric vehicles [5]. At the current stage, commercialized LIBs still rely on intercalation-type oxide cathodes and graphite anodes owing to their high capacity and lower cost [6]. The employed transition metal (TM) layered oxides with a hexagonal structure (space group *R* $\overline{3 }$ *m*) have remained as dominating cathode material over the past decades, including LiCoO_2_ [7] and LiNi_x_Co_y_Mn_z_O_2_ [8–11]. LiNi_x_Co_y_Mn_z_O_2_ (NCM) with high capacity, stable structure permits the utilization of the near-theoretical specific capacity of layered oxides in traditional lithium-ion battery structures [12]. However, long-time heat treatment has limited the application of NCM materials derived from the slow reaction dynamics of traditional synthesis methods, including solid-phase, sol–gel, and combustion [9, 13].

In comparison with traditional synthesis methods, high-temperature shock (HTS) technology can improve the synthesis efficiency and realize unique structural design of energy catalytic materials. For this reason, HTS method has been widely employed for ultrafast fabrication of nanoparticles, nanowires, graphene, etc. [14–17]. The degraded graphite anodes containing impurities can be rapidly upcycled by continuous high-temperature heating (≈2000 K) process, contributing to a high reversible capacity [18]. Recently, ultrafast HTS strategy has also been used to synthesize cathode materials, including LiMn_2_O_4_, LiCoO_2_, LiFePO_4_, and Li-rich layered oxide/NiO hetero-structured material [19]. Typically, the synthesis of NCM layered oxides cathodes requires a long-time heat treatment to produce fine crystalline structures, while the low heating rate makes the diffusion rate of ions slow, resulting in many non-equilibrium intermediate phases and sluggish reaction kinetics [20, 21]. Therefore, the rapid synthesis strategy breaks the thermodynamic/kinetic limitations of the conventional methods, improves the synthesis efficiency, and the ultrafast heating rate makes the cathode materials rapidly phased at high temperature, forming thermodynamically stable products.

Unfortunately, it is unclear how the ultrafast heating rate makes the ternary materials phased quickly and how long it takes to complete the phase transition during the HTS process. In this study, we successfully prepared a series of layered oxides cathodes (LiNi_x_Co_y_Mn_z_O_2_, NCM, x ≥ 0.5), and the approaching ultimate reaction rate of phase transition is calculated for the first time to explain the mechanism of rapid phase transition of NCM layered oxides. Furthermore, we first investigate the reaction process, in which the rapid heating rate brings fast solid reaction kinetic and the phase transition occurs rapidly. Precursors pass through the intermediate products quickly to generate the Li-containing oxides, and interestingly, the ultrafast average reaction rate is 66.7 (% s^−1^), taking only 1.5 s. Our research no longer solely furnished important points of segment evolution of NCM layered oxides during the HTS process but highlighted the significance of fast solid reaction kinetic, conducing to fine crystalline structures and high electrochemical performances. The findings are also expected to promote the commercialization and upscaling of Li-ion batteries by investigating the approaching ultimate phase transition rate of the solid-state calcination reaction.

## Experimental Procedures

### Synthesis of LiNi_x_Co_y_Mn_Z_O_2_ (x ≥ 0.5)

The precursors for NCM523, NCM622, and NCM811 with nominal composition were synthesized via a co-precipitation method. Specifically, an aqueous solution containing NiSO_4_·6H_2_O, CoSO_4_·7H_2_O, and MnSO_4_·H_2_O at a total concentration of 2.0 mol L^−1^ was pumped into a continuously stirring tank reactor under nitrogen at 55 °C. Simultaneously, a 10 wt% NaOH solution (as the precipitator) and a 5.0 mol L^−1^ NH_4_OH solution (as the chelating agent) were separately fed into the reactor. During the reaction, pH value of the system was controlled at 12.0–12.3. Upon the completion of the reaction, the resultant precursor precipitate was filtered and rinsed with deionized (DI) water. To obtain the layered oxides, the Ni_0.5_Mn_0.2_Co_0.3_(OH)_2_, Ni_0.6_Mn_0.2_Co_0.2_(OH)_2_, and Ni_0.8_Mn_0.1_Co_0.1_(OH)_2_ were first mixed with Li_2_CO_3_/LiOH·H_2_O by ball milling, and the nickel foil was used as a heating container. The mixtures were evenly spread on a nickel foil (2 cm × 5 cm), and the nickel foil-loaded mixtures were linked to a direct-current source with the current pulse by 90/210 s in HTS setup (Shenzhen Zhongkejingyan Company) and heated in air/O_2_. The temperature of the heater was tuned by adjusting the current and voltage and monitored by a laser infrared thermometer, and then the cathodes were obtained after thermal shock. The HTS process was carried out in air for NCM523, NCM622, and in oxygen for NCM811. The comparison samples were calcined in a tube furnace (TF).

### Characterization of Physical Properties

Crystalline phase structures of all materials were characterized by powder X-ray diffraction (XRD) with a Cu Kα radiation λ = 1.5406 Å, 40 kV, and 40 mA. Rietveld refinement was conducted using a GSAS-II code. High-resolution XRD patterns of synthesized materials were collected using a flat-panel X-ray detector at BSRF 3W1 station of the Institute of High Energy Physics of the Chinese Academy of Sciences. The wavelength of the X-ray beam used was 0.2061 Å. Morphologies and microstructures of all materials were investigated by scanning electron microscopy (SEM, S4800), transmission electron microscopy (TEM, JEM-2100F), and aberration-corrected TEM (JEM-ARM200F). N2 adsorption/desorption experiment was carried out for testing BET surface area (DX400). Average chemical compositions of cathode materials were analyzed by inductively coupled plasma-optical emission spectroscopy (ICP-OES Agilent 5110), while their cross-sectional compositions by electron probe microanalysis morphologies were conducted on focused ion beam (FIB, Crossbeam 350, ZEISS). Electron Energy Loss Spectroscopy (EELS) measurements were taken using the Themis Z microscope, equipped with a Gatan Quantum 977 spectrometer with a resolution of 0.1 eV/channel.

### Electrochemical Tests

Using a slurry procedure with N-methyl-2-pyrrolidone (NMP) as solvent and polyvinylidene fluoride (PVDF) as binder, all cathode materials were separately processed into slurries at a same composition of Active Material: Super-P: PVDF = 80: 10: 10 (wt%). The obtained slurries were spread onto Al foil current collectors, followed by drying at 80 °C under vacuum for 12 h, and the mass loading of the active material was ≈ 4.0 mg cm^−2^. Coin cells (CR2032) were assembled using lithium foils as counter electrodes, polypropylene separators (Celgard 2500, LLC Corp., USA), and electrolyte (CF4113C, Jiangsu Guotai). Galvanostatic charge–discharge (GCD) cycles were obtained by a mode of constant current/constant voltage for charge and constant current for discharge with a cut-off voltage of 2.8 ~ 4.3 V and different currents (1 C = 200 mA g^−1^).

## Results and Discussion

### Structural Evolution of the HTS Process

**Fig. 1:**
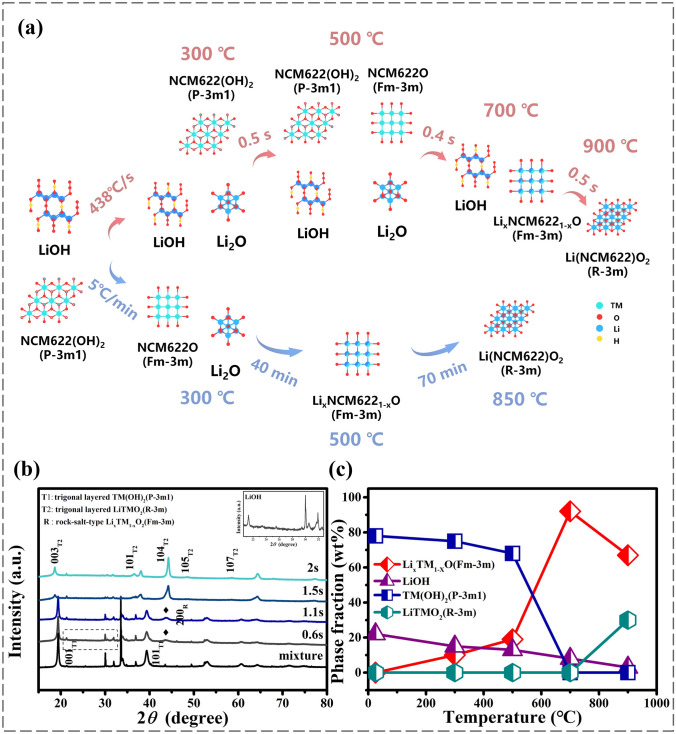
**a** Chemical and structural evolution of the HTS synthesis of LiNi_0.6_Co_0.2_Mn_0.2_O_2_ cathode materials. Comparison of the reaction paths of the HTS and conventional synthesis methods. **b** Ex situ XRD patterns of the mixture of TM(OH)_2_ and LiOH with heating at a rate of 438 °C s^−1^. The samples have been accumulated at distinctive temperatures following the heating profiles of quick synthesis of NCM622. **c** Quantitative evaluation of the weight fractions of exclusive phases got from Rietveld refinement of the corresponding XRD data

Figure 1a shows the compositional and structural evolution of the LiNi_0.6_Co_0.2_Mn_0.2_O_2_ cathode materials during the HTS process, combined with the XRD patterns shown in Fig. 1b. It is found that the diffraction intensity of lithium hydroxide was significantly reduced due to its rapid decomposition when the temperature rises rapidly from room temperature to 500 °C; meanwhile, the precursor Ni_0.6_Co_0.2_Mn_0.2_(OH)_2_ (CdI2-type, *P*$\overline{3 }$*m1*) undergoes a small decomposition reaction. The Bragg reflection of the Li-free rock salt-type intermediate NCM622O (*Fm*$\overline{3 }$*m*) near 43° appears at 300 °C, and the diffraction peak (200) becomes stronger after 300 °C. A large amount of layered CdI2-type structure (*P*$\overline{3 }$*m1*) is still retained in the product. Interestingly, Li-free rock salt-type intermediate NCM622O started to react with the lithium source to produce a Li-containing rock salt-type intermediate (Li_x_NCM622_1−x_O) within 0.4 s when the temperature rises from 500 to 700 °C. Then the Bragg reflection of the TM(OH)_2_ near 19.5° has shifted toward the (003) Bragg reflection of the *R*$\overline{3 }$*m* layered oxide at 18.5° at 700 °C [22]. The Li-containing rock salt-type intermediate gradually starts to convert to layered phase (*R*$\overline{3 }$*m*) after 700 °C, which can be verified by the intensification of (003) reflections. It is not difficult to find that there are more disordered rock salt structures in the products at 900 °C. Therefore, these observations demonstrate that the pristine TM(OH)_2_ undergoes the rapid phase transition into the Li-containing oxide from approximately 25–700 °C and the rapid transition occurs in 1.5 s, via the topotactic lithiation. Lithium ions from surface can move quickly into the interior of Li-free oxides, whereas some TM cations within the interior region are thought to be transported to the near-surface area by fast chemical reactions, forming the layered oxides [23]. The evolving phase fraction (Fig. 1c) was quantitatively assessed with the aid of outcomes of Rietveld refinements primarily based on the diffraction patterns. Evolution of lattice parameters determined from the Rietveld-refined XRD results is shown in Table S2. It is worth noting that a small amount of residual LiOH still existed at 700 °C, which may be attributed to the incomplete decomposition of lithium hydroxide in a very short time. These results proclaim that the precursors can be transformed into the layered structure in seconds during the HTS process. In contrast, the intermediary phases at each temperature are visible with traditional heating protocol. A low heating rate and sluggish reaction kinetic make the solid-state synthesis of NCM complicated, resulting in many non-equilibrium intermediate phases, including Li-free rock salt-type and Li-containing rock salt-type intermediates.

### Phase Transition Reaction Rate Calculation

**Fig. 2:**
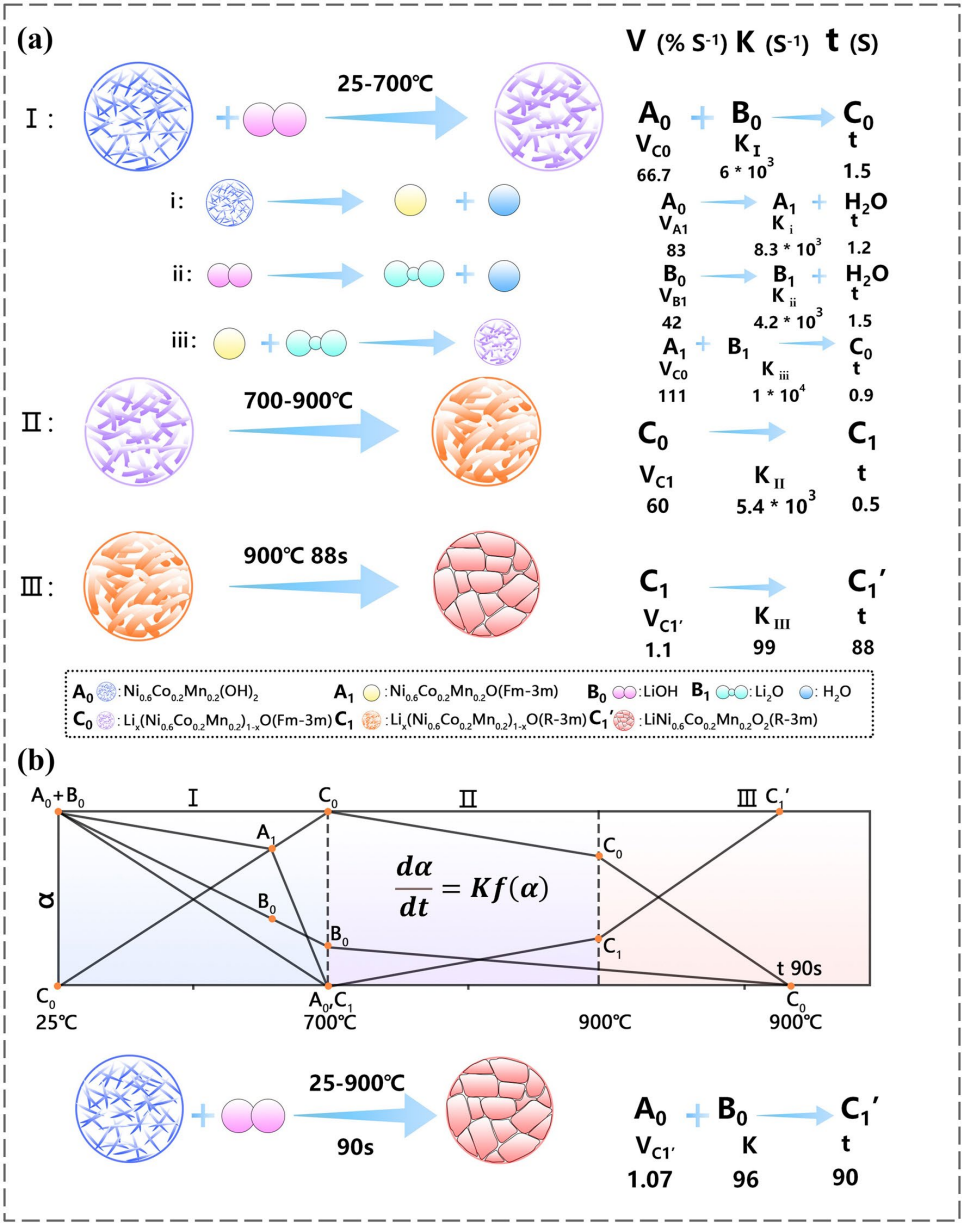
**a** Reaction model diagrams of the NCM622 cathode; the reaction rate at each stage is calculated and shown. **b** Schematic of the NCM622 cathode materials synthesis at different synthetic temperatures and holding times: Low temperature (25 ~ 700 °C; I) completed the phase transition from layered TM(OH)_2_ to intermediate Li_x_NCM622_1−x_O; optimized temperature (700 ~ 900 °C; II) enables the formation of layered oxides with structural disordering; and holding times (~ 900 °C; III) cause particle growth and perfect layered crystal structure

In order to quantify the reaction rate of phase transition from the precursor Ni_0.6_Co_0.2_Mn_0.2_(OH)_2_ to intermediate Li_x_NCM622_1−x_O, to layered structured LNCM622O during heat treatment, we use the differential of the variable temperature rate equation to describe the variable temperature solid reaction rate, conversion, and temperature functional relationship (Fig. 2a). Generally, under the linear heating condition, the kinetic equation can be expressed as: $\frac{{\text{d}}\alpha }{{\text{d}}t}={{\text{Ae}}}^{-\frac{E}{\hbox{RT}}}f\left(\alpha \right)=Kf(\alpha )$, where $f(\alpha )$ is the function of reaction mechanism, $\alpha$ is the degree of conversion in reaction, $T$ is the temperature, $A$ is the pre-exponential factor, $\beta$ is the linear heating rate ($\beta ={\text{d}}T/{\text{d}}t$), $R$ is the gas constant, and $E$ is the activation energy [24]. Meanwhile, the relationship between the conversion rate $\alpha$ and temperature T can be expressed as: $\frac{\hbox{d}\alpha }{\hbox{d}T}=\frac{\hbox{d}\alpha }{\hbox{d}t}\cdot \frac{\hbox{d}t}{\hbox{d}T}=\frac{Kf(\alpha )}{\beta }$. The average chemical reaction rate is approximated by the change in conversion per unit time: V_average_ $=$ $\frac{\hbox{d}\alpha }{\hbox{d}t}$. Meanwhile, the reaction rate constant *K* is also used to describe how fast or slow the reaction is, and *K* is temperature dependent (*K* $= \frac{\frac{\hbox{d}\alpha }{\hbox{d}T}\cdot \beta }{f(\alpha )}$). Here, $\alpha , t$ is substituted according to the change in phase content shown in Fig. 1b, c. At a low synthesis temperature of 700 °C, the presence of layered TM(OH)_2_ has not been observed, indicating that ultrafast average reaction rate of phase transition from Ni_0.6_Co_0.2_Mn_0.2_(OH)_2_ to intermediate Li_x_NCM622_1−x_O is 66.7% s^−1^ ($\alpha =100\%, t=1.5 \, {\text{s}}$). This solid-state reaction kinetic has been described by diffusion models, that is, $f\left(\alpha \right) = {[1-{\left(1-\alpha \right)}^\frac{1}{3}]}^{-1}\cdot {\left(1-\alpha \right)}^\frac{2}{3}$. Interestingly, layered TM(OH)_2_ decomposes to form Li-free rock salt-type intermediate NCM622O, and the decomposition of LiOH has been observed during the heating process from 25 to 700 °C, in which the decomposition reaction kinetics have been described by nucleation model, that is, $f\left(\alpha \right) = {\left(1-\alpha \right)}^\frac{2}{3}$. More importantly, the intermediate product reacts with lithium source to quickly form Li-containing rock salt-type intermediate Li_x_NCM622_1−x_O (A_1_ + B_1_ → C_0_), in which the reaction is the speed control step. Therefore, relatively fast rate of warming accelerates chemical reactions and phase transition. On the other hand, an increase in the synthesis temperature up to 900 °C accelerates the formation of layered oxides (average reaction rate: 60% s^−1^) with structural disordering. Structural ordering has also been improved with the extension of holding time (to be discussed below). Figure 2b shows the NCM622 cathode materials synthesis at different synthetic temperatures and holding times. These results indicate that ultrahigh heating rate makes fast reaction kinetics and induces the rapid phase transition of NCM cathodes; meanwhile, precursors pass through the intermediate products quickly to generate a good layered structure, taking only a few tenths of a second.

### Morphology and Structure Characterization

**Fig. 3:**
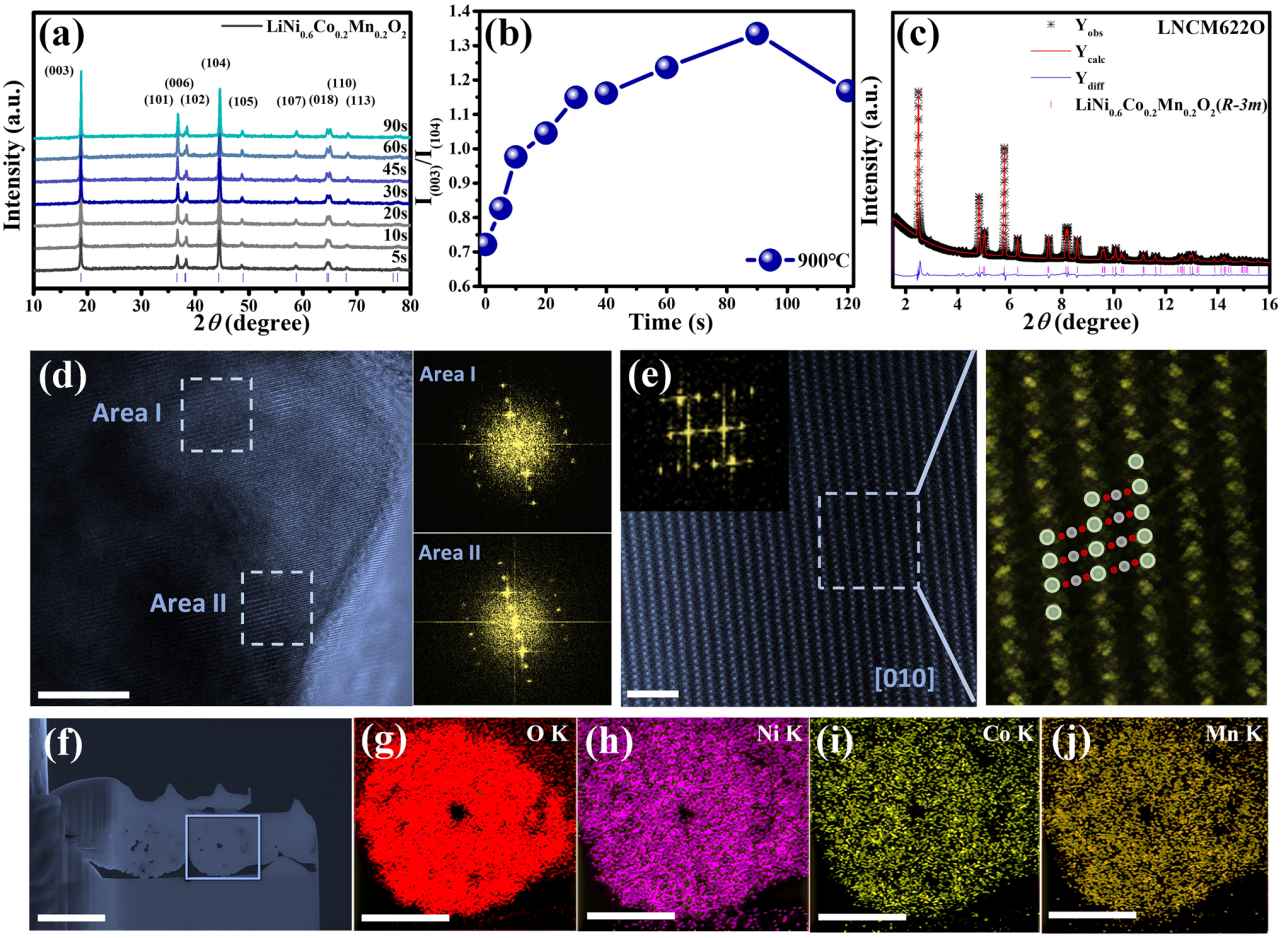
Synthesis, structural characterization of the synthesized LiNi_0.6_Co_0.2_Mn_0.2_O_2_ cathode materials. **a** Ex situ XRD patterns evolution of NCM622 in the HTS process at 900 °C. **b** Evolution of the cationic disordering. **c** Refined X-ray diffraction pattern of 900 °C, 90-s sample. The sample consists of the layered *R*$\overline{3 }$*m* (Li–TM–O_2_). **d** TEM image for 900 °C, 90-s sample, scale bar, 10 nm. The inset images are the fast Fourier transform pattern for the TEM image. **e** HAADF-STEM image, scale bar, 2 nm, corresponding atomic mode. **f**–**j** FIB image, scale bar, 2.5 μm and EDX elemental mapping of O, Ni, Co, and Mn cross-section of secondary particle for pristine NCM622 cathode, scale bar, 1 μm

Figure 3a shows the XRD patterns recorded from intermediates of NCM622 at 900 °C in air. Corresponding XRD patterns of NCM523 and NCM811 are presented in Figs. S1 and S2. It is found that the layered structure was already formed in 5 s at the target temperature. The improvement of structural ordering is demonstrated by the continued rise in peak intensity in the (003) and (104) reflections (Fig. 3b), as well as the increased splitting of the (018)/(110) peaks, and increasing lattice parameters c/a ratio (Fig. S3). Meanwhile, these peaks narrowed over time, demonstrating the increase in crystallinity following heat treatment. A similar variation trend was observed in NCM523 and NCM811 (Figs. S1–S4), except that NCM811 needs a lengthy preserving time to make the oxidation of Ni in the fabric [20, 25, 26]. In addition, the usual morphology of secondary particles, with a measurement of around 3 µm (shown by way of SEM photographs in Fig. S7), was once maintained in the course of the HTS process. Compared with the Ni_0.6_Co_0.2_Mn_0.2_(OH)_2_ precursor, this calcination step largely maintains the spherical morphology. With the extension of holding times, the primary particles grow obviously larger, regardless of the aggregation forming secondary spheres. It is evident from Fig. S8 that the primary particles inside the secondary particles also grow, so such a short time can keep a consistency between the internal and surface morphology of the particles. Corresponding SEM images of NCM523 and NCM811 are presented in Figs. S9 and S10. The specific surface area values of the final samples NCM622 are presented in Fig. S11 and Table S3. Rietveld refinement was once carried out on HEXRD patterns acquired from the 900 °C, 90-s sample, which is consisted of the layered *R*$\overline{3 }$*m* (Fig. 3c)*.* Similar results of NCM523 and NCM811 (Fig. S6) demonstrate good layered structures after a short-time heat treatment. The TEM characterization further confirms that the layered phase was uniformly formed in the HTS-synthesized NCM622, NCM523, and NCM811 (Figs. 3d and S12, S13). In addition, the crystal lattice of NCM622 was further illustrated by means of the corresponding atomic model of the HAADF-STEM image (Fig. 3e). Oxygen ions are cubic packed tightly to form octahedron, where Li^+^ and TM^n+^ are alternately located in the octahedral position of the dense oxygen layer [6, 27]. Figure 3f–j presents cross-sectional scanning electron microscopy images of NCM622 cathode materials and EDS elemental maps of O, Ni, Co, and Mn, indicating that the elements in the positive electrode of NCM622 synthesized by HTS technology in a short time are evenly distributed. EELS mapping is utilized to characterize the distribution of lithium elements (Fig. S14). It is evident from the figures that the lithium sources are well dispersed and evenly distributed inside the particle within a few seconds during HTS. The chemical compositions of the rapidly synthesized NCM523, NCM622, and NCM811 cathodes are presented in Table S1.

### Battery Performance

**Fig. 4:**
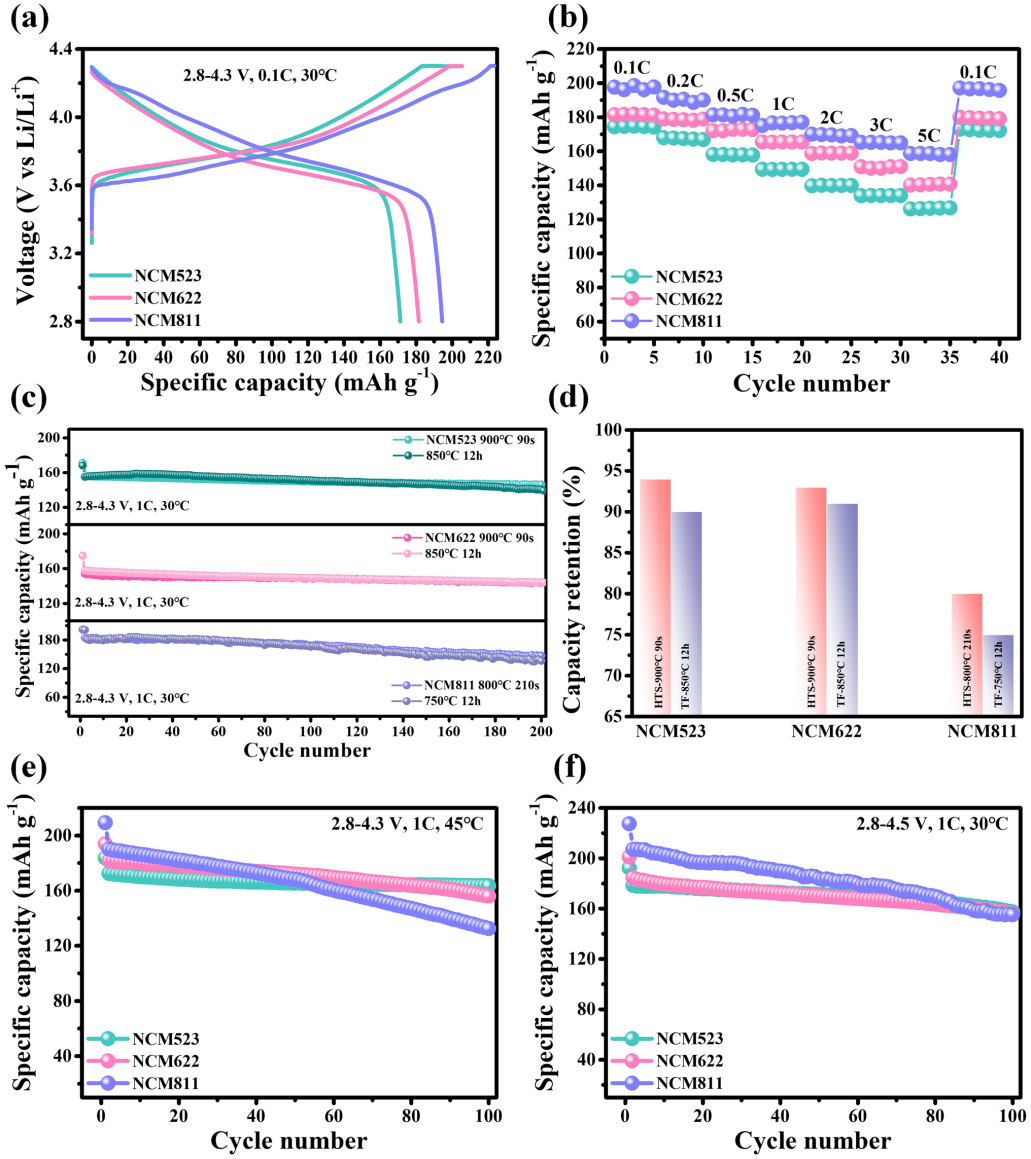
**a** Initial charge and discharge voltage profiles at 2.8–4.3 V, **b** rate capability, and **c** cycling performances at 30 °C for the NCM523, NCM622, and NCM811 cathodes. **d** Summary of capacity retention after 200 cycles. The cyclic performances of traditional methods are in contrast. **e** Cycling performances at 45 °C and **f** 2.8–4.5 V for these cathodes

The fundamental electrochemical performances of three HTS-synthesized cathodes were evaluated. Figure 4a shows the initial charge and discharge profiles at 2.8–4.3 and 2.8–4.5 V. At 4.3 V, NCM523, NCM622, and NCM811 delivered a discharge capacity of 172, 181, and 195 mAh g^−1^ at 0.1 C, respectively. Accordingly, they delivered the discharge capacities of 195, 201, and 226 mAh g^−1^, respectively, under 0.1 C and 4.5 V (Fig. S17). At 4.3 V, the capacity retention after 200 cycles regularly reduced with increasing the Ni fraction (94% for NCM523, 94% for NCM622, and 80% for NCM811) (Fig. 4c, d). The cycling performance is nearly the same as traditional methods. The capacity retention after 100 cycles at 4.5 V differs little from long calcined cathodes (87% vs. 81% for NCM523, 85% vs. 81% for NCM622, and 75% vs. 71% for NCM811) (Figs. 4f and S18). The stabilization of the lithiated shape led to a most suitable stability, which is, in addition, accentuated at a greater voltage. One of the quintessential standards for a cathode used in an EV is its potential to hold an environment-friendly overall performance over a broad temperature range. Based on electrochemical, structural, and mechanical balance outcomes, the cycling performances of the NCM523, NCM622, and NCM811 cathodes have been examined at 45 °C (Fig. 4e and charge and discharge curves in Fig. S15). NCM523, NCM622, and NCM811 retained 95%, 86%, and 70% of its initial capacity after 100 cycles at 1 C, 45 °C, respectively, and the high-temperature performance of NCM811 with high nickel composition needs to be further improved (Fig. S16). Rate capability is also one of the key performance indicators of power battery cathodes, and rate performances of three cathodes are shown in Fig. 4b. Three cathodes provide the reversible discharge capacity of 130, 140, and 160 mAh g^−1^ at 5 C, respectively. Three layered oxides cathodes by the HTS-synthesized strategy have good electrochemical properties. All cycling stability comparisons are shown in Table S4.

## Conclusion

In this study, rapid synthesis strategy has been successfully applied to prepare a series of ternary layered cathode materials in a short time, breaking the thermodynamic/kinetic limitations of the conventional methods. The results from quantitative structure analysis showed that the ultrafast heating rate makes the precursors rapidly phased and precursors can pass through the intermediate products quickly, forming thermodynamically stable products after a short holding time. Furthermore, one thing is for sure that the reaction rate of intermediate transition to layered structure is fast, and lithium ions and TM cations quickly transfer to form a good layered structure during heat treatment. The layered oxides LiNi_x_Co_y_Mn_z_O_2_ are prepared through rapid synthesis and perform good cycling performances (94% for NCM523, 94% for NCM622, and 80% for NCM811 after 200 cycles at 4.3 V). The HTS strategy in this study may open a new avenue for kinetic control of the reaction pathway, and fast solid reaction kinetics can be simultaneously achieved at a high heating rate. Finally, these findings provide new insights into solid phase transformation rate for layered oxides LiNi_x_Co_y_Mn_z_O_2_.

## Supplementary Information

Below is the link to the electronic supplementary material.

Supplementary file1 (DOCX 39564 kb)
