# Supplementary material for: Approaching Ultimate Synthesis Reaction Rate of Ni-Rich Layered Cathodes for Lithium-Ion Batteries
Source: Nanomicro Lett. 2024 Jun 6;16:210. doi: 10.1007/s40820-024-01436-y (PMC11156821; doi:10.1007/s40820-024-01436-y)
Supplement: Supplementary file 1 — Supplementary file1 (DOCX 39564 kb) [file 40820_2024_1436_MOESM1_ESM.docx]

Supporting Information for

**Approaching Ultimate Synthesis Reaction Rate of Ni-Rich Layered Cathodes for Lithium-Ion Batteries**

Zhedong Liu^1^, Jingchao Zhang^1^, Jiawei Luo^1^, Zhaoxin Guo^1^, Haoran Jiang^1^, Zekun Li^1^, Yuhang Liu^1^, Zijing Song^1^, Rui Liu^2^, Wei-Di Liu^3^, Wenbin Hu^1,^ *, Yanan Chen^1,^ *

^1^ School of Materials Science and Engineering, Tianjin University, Tianjin 300072, P. R. China

^2^ School of Materials Science and Engineering, Shandong University of Science and Technology, Qingdao 266590, P. R. China

^3^Australian Institute of Bioengineering and Nanotechnology, The University of Queensland, St Lucia, QLD, Australia 4072

*Corresponding authors. E-mail: [yananchen@tju.edu.cn](mailto:yananchen@tju.edu.cn) (Yanan Chen); [wbhu@tju.edu.cn](mailto:wbhu@tju.edu.cn) (Wenbin Hu)

**Supplementary Figures and Tables**

**Fig. S1** (**a**) Ex situ XRD patterns evolution of NCM523 in the HTS process at 900 ℃. (**b**) Evolution of the cationic disordering

**Fig. S2** (**a**) Ex situ XRD patterns evolution of NCM811 in the HTS process at 800 ℃. (**b**) Evolution of the cationic disordering

**Fig. S3** Evolution of lattice parameters a, c and the c/a ratio with holding time during synthesis of NCM622 at 900 ºC

**Fig. S4** Evolution of lattice parameters a, c and the c/a ratio with holding time during synthesis of NCM523 at 900 ºC

**Fig. S5** Evolution of lattice parameters a, c and the c/a ratio with holding time during synthesis of NCM811 at 800 ºC

**Fig. S6** (**a**) Refined X-ray diffraction pattern of NCM523 sample (900 °C 90 s) and NCM811 sample (800 °C 210 s). (**b**) Rietveld refinements based on layered phase were carried out and the samples consist of the layered *R*$\bar{3}$*m* (Li–TM–O_2_)

**Fig. S7** (**a**) The calcination temperature curve of NCM622 at 900 °C for 90 s. (**b**) SEM images of NCM622 samples sintered under the conditions of (c) 900 ℃-0 s, (**d**) 10 s, (**e**) 20s, (**f**) 60 s, (**g**) 90 s and (**h**) 120 s, scale bar, 1 μm

**Fig. S8** (**a**) SEM images of NCM622 at 900 °C for 90 s, scale bar, 1 μm. (**b, c**) FIB images, scale bar, 500 nm. (**d**) Enlarged image of internal morphology of the particles, scale bar, 200 nm


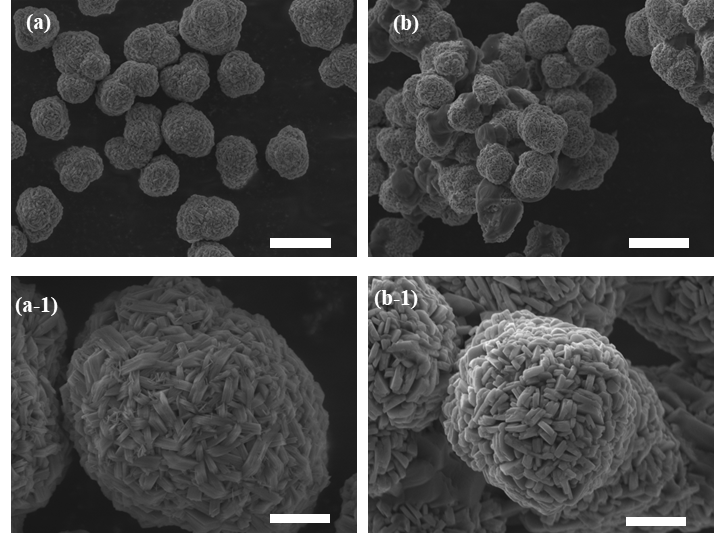


**Fig. S9** (**a**), (**a-1**) SEM images of precursors, scale bar, 5 μm, 1 μm and (**b**), (**b-1**) NCM523 samples sintered under the condition of 900 ℃-90 s, scale bar, 5 μm, 1 μm


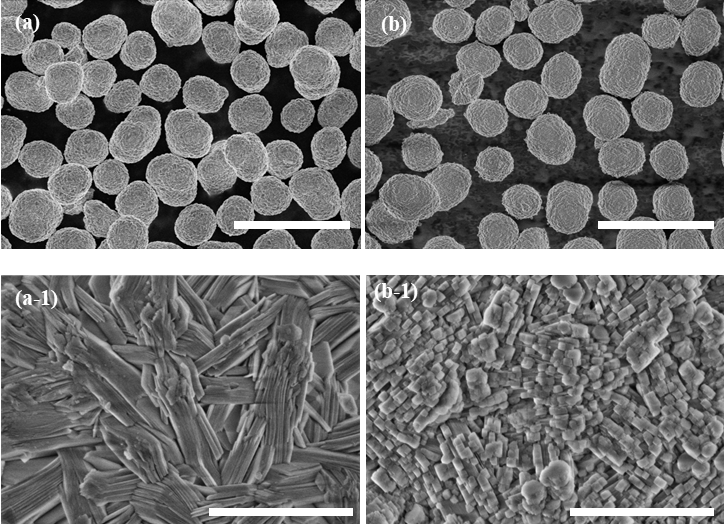


**Fig. S10** (**a**), (**a-1**) SEM images of precursors, scale bar, 20 μm, 1 μm and (**b**), (**b-1**) NCM811 samples sintered under the condition of 800 ℃-210 s, scale bar, 20 μm, 1 μm

**Fig. S11** N_2_ adsorption and desorption curves of HTS-NCM622 and TF-NCM622

**Fig. S12** TEM image for NCM523 (900 °C 90 s) sample, scale bar, 2 nm. The inset images are the fast Fourier transform pattern for the TEM image, scale bar, 2 μm

**Fig. S13** TEM image for NCM811 (800 °C 210 s) sample, scale bar, 10 nm. The inset images are the fast Fourier transform pattern for the TEM image, scale bar, 10 μm

**Fig. S14** (**a**) FIB-image of NCM622 samples, scale bar, 1 μm. (**b**) Enlarged image of internal morphology of the particles, scale bar, 200 nm. (**c**) EELS-Mapping of lithium, scale bar, 200 nm

**Fig. S15** Initial charge and discharge voltage profiles of LiNi_x_Co_y_Mn_z_O_2_ at 45℃, 2.8–4.3 V

**Fig. S16** Cycle performances at 45 °C, 2.8-4.3V for the NCM523, NCM622, and NCM811 cathodes obtained using 2032-type coin cells with Li metal as a counter electrode. The cyclic performances of traditional methods are in contrast. (1C = 200 mA g^−1^)

**Fig. S17** Initial charge and discharge voltage profiles of LiNi_x_Co_y_Mn_z_O_2_ at 30 ℃, 2.8–4.5 V

**Fig. S18** Cycle performances at 30 °C, 2.8-4.5V for the NCM523, NCM622, and NCM811 cathodes obtained using 2032-type coin cells with Li metal as a counter electrode. The cyclic performances of traditional methods are in contrast. (1C = 200 mA g^−1^)

**Table S1** Chemical compositions of the rapid synthesized NCM523, NCM622, NCM811 cathodes

| **Sample** | **ICP-AES (wt%)** | | | |
| --- | --- | --- | --- | --- |
|  | **Li** | **Ni** | **Co** | **Mn** |
| NCM523 | 7.19 | 30.39 | 12.21 | 17.99 |
| NCM622 | 7.16 | 36.33 | 12.16 | 11.94 |
| NCM811 | 7.13 | 48.27 | 6.06 | 5.95 |
| **Sample** | **Measured molar ration** | | | |
|  | **Li** | **Ni** | **Co** | **Mn** |
| NCM523 | 1.036 | 0.492 | 0.197 | 0.311 |
| NCM622 | 1.032 | 0.594 | 0.198 | 0.208 |
| NCM811 | 1.027 | 0.796 | 0.099 | 0.105 |

**Table S2** Structural characteristics were determined from the Rietveld-refined XRD results at different temperatures following the heating profiles of fast synthesis of NCM622

| **Material** | **a (Å)** | **c (Å)** | **c/a** |
| --- | --- | --- | --- |
| precursor | 3.0543 | 4.5660 | 1.494 |
| 300℃ | 3.0510 | 4.5689 | 1.497 |
| 500℃ | 3.0482 | 4.5793 | 1.502 |
| 700℃ | 2.8983 | 14.2812 | 4.927 |
| 900℃ | 2.8855 | 14.2285 | 4.931 |

**Table S3** The specific surface area test results

|  | HTS-NCM622 | TF-NCM622 |
| --- | --- | --- |
| BET (m^2^/g) | 0.7797 | 0.8125 |

**Table S4** Comparison of cycling stability of LiNi_x_Co_y_Mn_z_O_2_ cathodes with the Ni-rich based cathodes reported before for half cells at 30 ℃

| **Sample** | **1^st^ capacity**  **(mAh g^-1^)** | **Retention** | **References** |
| --- | --- | --- | --- |
| NCM523 | 172 | 94%（2.8-4.3V, 1C, 200 cycles） | This work |
| NCM622 | 181 | 94%（2.8-4.3V, 1C, 200 cycles） | This work |
| NCM811 | 195 | 80%（2.8-4.3V, 1C, 200 cycles） | This work |
| NCM523 | 173 | 96.9%（3-4.3V, 0.5C, 100 cycles） | [S1] |
| NCM622 | 185 | 94%（3-4.3V, 0.5C, 100 cycles） |  |
| NCM811 | 204 | 81%（3-4.3V, 0.5C, 100 cycles） |  |
| SrCO_3_-NCM523 | 163 | 90%（2.8-4.3V, 1C, 100 cycles） | [S2] |
| ZrB_2_-NCM811 | 202 | 90%（3-4.3V, 1C, 100 cycles） | [S3] |
| RHSNCM523 | 155(0.5C) | 92%（2.5-4.3V, 0.5C, 100 cycles） | [S4] |
| RHSNCM622 | 167(0.5C) | 90%（2.5-4.3V, 0.5C, 100 cycles） |  |
| Li_2_BO_3_flux-NCM523 | 175 | 80%（2.8-4.3V, 1C, 100 cycles） | [S5] |
| NCM523-IE200-PL800 | 170.3 | 86.2%（2.7-4.3V, 1C, 200 cycles） | [S6] |
| NCM811-BE-LiHMDS | 182(0.5C) | 73.9%（3.0-4.5V, 0.5C, 1000 cycles） | [S7] |
| NCM811-FETT | 200(0.5C) | 68%（3.0-4.6V, 0.5C, 150 cycles） | [S8] |
| NCM811 | 195.2 | 72.4%（2.7-4.3V, 0.5C, 200 cycles） | [S9] |

**Supplementary References**

1. H.-J. Noh, S. Youn, C. S. Yoon, Y.-K. Sun, Comparison of the structural and electrochemical properties of layered Li[Ni_x_Co_y_Mn_z_]O_2_ (x=1/3, 0.5, 0.6, 0.7, 0.8 and 0.85) cathode material for lithium-ion batteries. J. Power Sources **233**, 121-130 (2013). <https://doi.org/10.1016/j.jpowsour.2013.01.063>
2. G. Hu, L. Li, Y. Lu, Y. Cao, Z. Peng et al., SrCO_3_ assisted synthesis of disk-like micron-sized monocrystalline LiNi_0.5_Co_0.2_Mn_0.3_O_2_ with Preferred (104) plane and its enhanced cycle performance. J. Electrochem. Soc. **167**, (2020). <https://doi.org/10.1149/1945-7111/abc032>
3. Z. Feng, R. Rajagopalan, S. Zhang, D. Sun, Y. Tang et al., A three in one strategy to achieve zirconium doping, boron doping, and interfacial coating for stable LiNi(0.8)Co(0.1)Mn(0.1)O(2) Cathode. Adv. Sci. **8**, 2001809 (2021). <https://doi.org/10.1002/advs.202001809>
4. K. Jia, J. Wang, Z. Zhuang, Z. Piao, M. Zhang et al., Topotactic Transformation of Surface Structure Enabling Direct Regeneration of Spent Lithium-Ion Battery Cathodes. J. Am. Chem. Soc. **145**, 7288-7300 (2023). <https://doi.org/10.1021/jacs.2c13151>
5. T. Yamada, K. Shishino, Y. Doya, K. Fujisawa, K. Teshima, Individual effects of flux species as a reaction field on coprecipitation precursor toward the design of fine, mono-dispersed LiNi_0.5_Co_0.2_Mn_0.3_O_2_ single crystals. ACS Appl. Energy Mater. **6**, 245-256 (2022). <https://doi.org/10.1021/acsaem.2c02884>
6. Y.-h. Luo, Q.-l. Pan, H.-x. Wei, Y.-d. Huang, L.-b. Tang et al., Regulating cation mixing for enhanced structural stability of layered oxide cathodes by ion-exchange strategy. Mater. Today **69**, 54-65 (2023). <https://doi.org/10.1016/j.mattod.2023.08.006>
7. J. Huang, Y. Yang, Y. Liu, J. Ma, Lithium Hexamethyldisilazide Endows Li||NCM811 Battery with Superior Performance. Nano-Micro Lett. **15**, 33 (2023). <https://doi.org/10.1007/s40820-022-00998-z>
8. J. Liu, X. Li, J. Huang, G. Yang, J. Ma, Additive‐guided solvation-regulated flame-retardant electrolyte enables high-voltage lithium metal batteries with robust electrode electrolyte interphases. Adv. Funct. Mater. 2312762 (2024). <https://doi.org/10.1002/adfm.202312762>
9. Y. Han, Y. Zhang, Y. Lei, D. Xiao, J. Ni et al., Regulating cathode-electrolyte interphase by confining functional aluminum compound within Ni-rich cathodes. Adv. Funct. Mater. **33**, 2301642 (2023). <https://doi.org/10.1002/adfm.202301642>
